# Evaluation of 3D Trapezoidal Plates in Open Reduction and Internal Fixation of Subcondylar Fractures of Mandible: A Clinical Trial

**DOI:** 10.7759/cureus.15537

**Published:** 2021-06-08

**Authors:** Triveni Palani, Srimathi Panchanathan, Davidson Rajiah, Arunkumar Kamalakaran, Abdul A Hafeez, Priyadharshini Raghavan

**Affiliations:** 1 Department of Oral and Maxillofacial Surgery, Tamilnadu Government Dental College and Hospital, Chennai, IND; 2 Department of Oral and Maxillofacial surgery, Tamilnadu Government Dental College and Hospital, Chennai, IND

**Keywords:** mandibular fractures, subcondylar fractures, three-dimensional (3d) plates, open reduction and internal fixation (orif)

## Abstract

Background

Fractures of the mandibular condyle are common and account for 25% to 50% of all fractures of the mandible. Various methods exist for open reduction and internal fixation (ORIF) of condylar fractures. This study was done to explore three-dimensional (3D) plates as a viable option.

Aim

This study aims to evaluate the effectiveness of 3D trapezoidal plates in open reduction and internal fixation of subcondylar fractures.

Materials and methodology

This was a non-randomized clinical trial conducted on 20 patients who reported at the Department of Oral and Maxillofacial Surgery, Tamil Nadu Government Dental College and Hospital, Chennai, India. The ORIF was done under general anesthesia. A retromandibular transmasseteric approach was used to expose the fracture site, and the fracture was stabilized using 3D titanium trapezoidal plates. Parameters, such as mouth opening, mandibular deviation, occlusion, surgical accessibility, fracture reduction, adaptability, the difference in ramal height, angulation of the fractured condyle, operative time, facial nerve weakness, implant failure, complications, and scar formation were assessed. Statistical analysis was done using Statistical Package for Social Sciences (SPSS), version 21 (IBM SPSS Statistics for Windows, Armonk, NY).

Results

There was an improvement in mouth opening and occlusion in the immediate postoperative period. The surgical accessibility ranged from good to excellent. The fracture reduction was excellent in 60% of patients and good in 40%. In one patient, there was a transient weakness of the marginal mandibular nerve which recovered by three months. Another patient had a wound infection that subsided within the first postoperative week. None of the patients had a device failure during the six-month follow-up period.

Conclusion

The trapezoidal 3D plates could be considered as a viable option for treating subcondylar fractures of the mandible in terms of surgical accessibility, stability, ease of device placement, stability of reduced fracture, reduced osteosynthesis material requirement, and minimal damage to the surrounding tissues.

## Introduction

Trauma to the face causes injuries to skeletal components, soft tissue, and dentition as well. The incidence and pattern of fractures varies in different regions depending on geographical, cultural, environmental, and social attribute [1]. Road traffic accidents are the most common cause of maxillofacial trauma in developed nations; however, interpersonnel violence is the major cause in developing countries and the Western world [2]. Due to its prominent position, the mandible is often involved in maxillofacial trauma, contributing to about 65% - 70% of facial fractures [3]. In 2001, Haug and Assael reported an incidence of 30.3% for condylar fractures [4]. In maxillofacial trauma, treatment for condylar fractures is still an ongoing controversy.

The three main treatments advocated for adult condylar fractures are closed reduction with maxillo-mandibular fixation (MMF) followed by functional rehabilitation, functional therapy without MMF, and open reduction with/without MMF. Owing to its complex anatomy, biomechanical behavior, and extraordinary healing potential, any injury of the condyle deserves special consideration. According to recent concepts, fractures with a deviation of more than 10° or a shortening of the ascending ramus of more than 5 mm should be treated with open reduction and fixation, regardless of the level of the fracture [5]. The condyle is a complex, anisotropic, and viscoelastic material subjected to different types of strain (tension, compression, bending, or shearing) on loading. It raises the problem of finding a fixation device that can resist local strains, adapting to the anatomical and functional peculiarities of the region, providing easy surgical access, and being cost-effective to the patient. Also, a miniature osteosynthesis device becomes essential for the stabilization of subcondylar fractures because of the usually small size of the condylar fragments. It is mandatory to place these plates along "the ideal line of osteosynthesis" for dictating a predictable outcome. In 1976, Champy et al. experimentally located these strain lines in the mandibular body, symphysis, and angle region [6]. In 2002, Meyer et al. proposed the ideal lines of osteosynthesis in the condylar region [7].

The widely accepted technique for osteosynthesis of subcondylar fractures has been the double miniplate technique. However, studies have documented a failure rate of 35%, including plate fracture. In 1995, Farmand developed titanium 3D plating systems to meet the requirements of semi-rigid fixation with lesser complications [8]. The word 3D is a misnomer as the plate is not three-dimensional but resists the forces in three directions, namely, bending, shearing, and torsion. The basic concept of 3D fixation is that a geometrically closed quadrangular mini-plate is secured with the bone. Trapezoidal condylar plates (3D plates) were developed to meet these biofunctional demands in the condylar region. The purpose of this study was to evaluate the effectiveness of the trapezoidal plates (3D plates) in open reduction and internal fixation of subcondylar fractures of the mandible.

## Materials and methods

Study design

This study is an uncontrolled non-randomized clinical trial which was conducted on 20 patients who reported to the Department of Oral and Maxillofacial Surgery, Tamil Nadu Government Dental College and Hospital from January 2016 to December 2017. Approval for this study was granted by the Institutional Ethics Committee (IEC approval number: RCNo0430/DE/2015 dated March 10, 2015).

Sample recruitment

**Figure 1:**
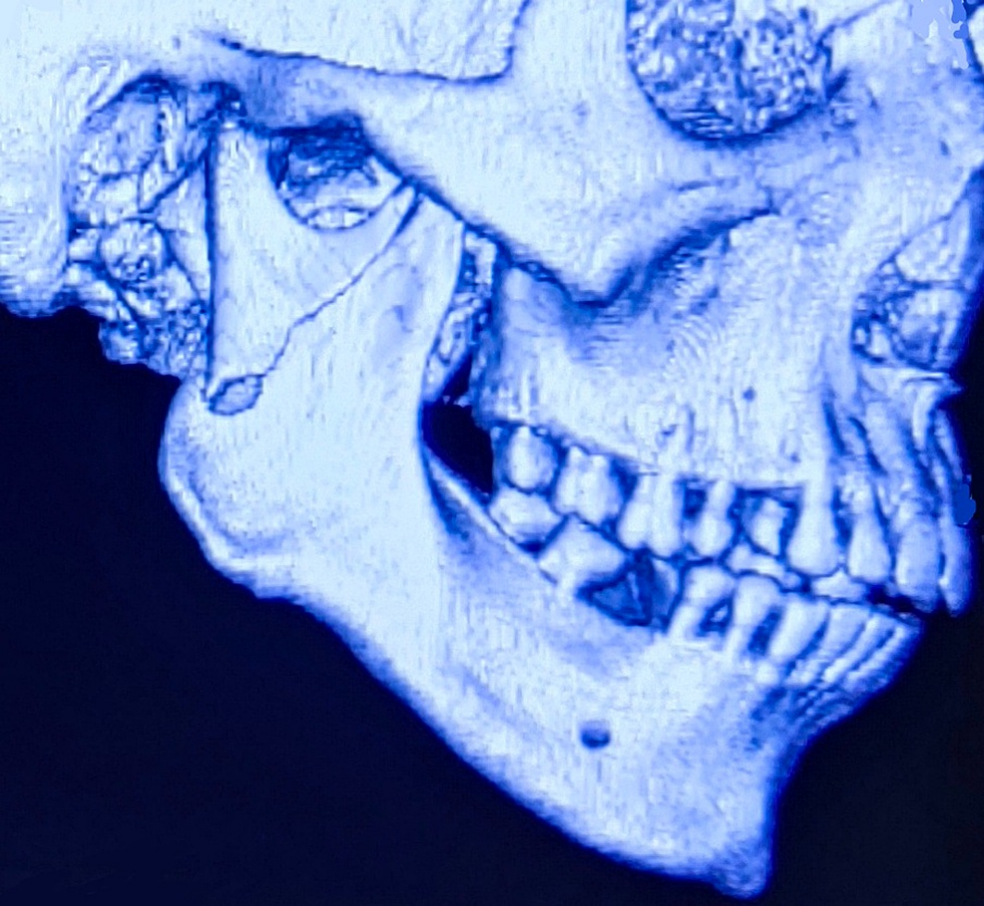
Type II condylar fracture according to the Spiessl and Schroll classification (Patient 1)

Convenient sampling was done for the recruitment of patients. The patients were recruited based on the inclusion and exclusion criteria, and consent was obtained from the patients before the start of the study. Included in the study were dentulous patients, 15 to 40 years old, and those who sustained simple/compound, unilateral subcondylar fractures with deranged occlusion associated with or without other facial fractures. All patients with a type II condylar fracture according to the Spiessl and Schroll classification were included in the study [9]. Patients with high condylar fractures, bilateral subcondylar fractures, comminuted condylar fractures, pediatric condylar fractures, pathologic fractures, edentulous patients, immunocompromised patients, and those with systemic disorders were excluded. The open reduction and internal fixation (ORIF) procedure was performed within one week of trauma, and all cases were operated on by the same chief surgeon. The procedure to be performed was explained and an information sheet was provided to all recruited patients. For each patient, a detailed history followed by a thorough clinical examination was done. Orthopantomogram (OPG) and a computerized tomography (CT) scan in all three planes with 3D reconstruction were done and findings were recorded in a specially prepared case history chart (Figure 1). All routine preoperative workup was done. Preoperatively, the degree of displacement was measured using the OPG. Postoperatively, the patients were followed up for six months at regular intervals to assess parameters.

Parameters assessed

The parameters assessed were mouth opening, mandibular deviation, occlusion, surgical accessibility and plate adaptability, reduction of fracture fragments, wound infection, facial nerve weakness, scar, postoperative pain, and osteosynthesis device failure.

Surgical technique

**Figure 2:**
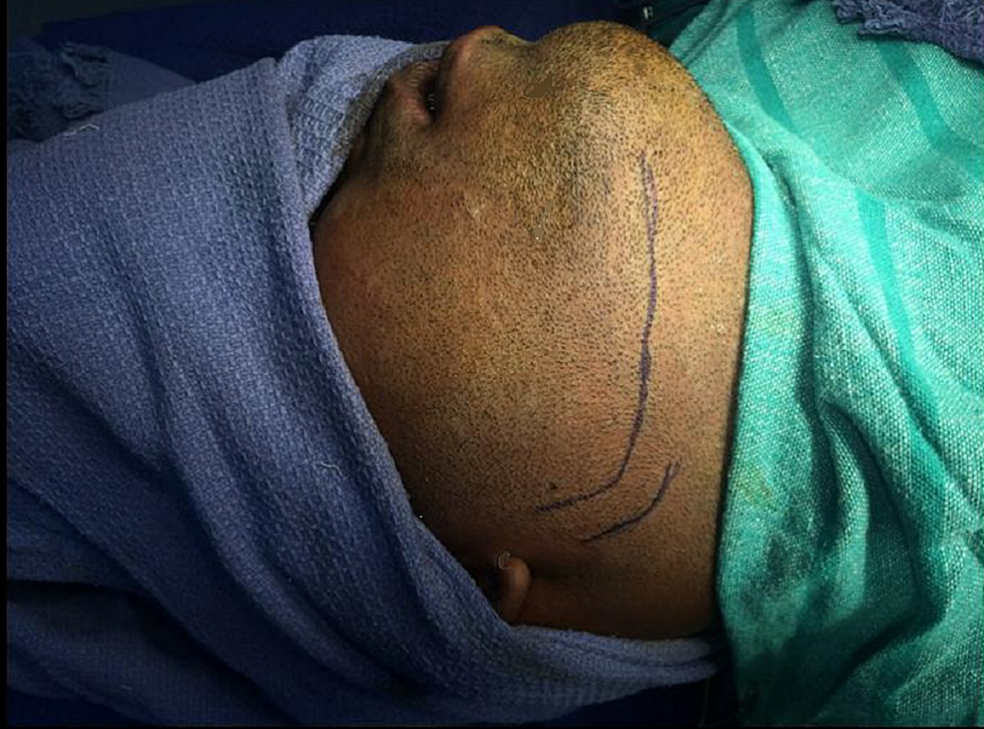
Surgical marking of retromandibular incision for transmasseteric approach to expose fractured subcondyle (Patient 1)

After ruling out head and cervical spine injury, cases were planned for surgery based upon the clinical and radiographic assessment. After oral prophylaxis, Erich arch bars were placed. All patients were treated under general anesthesia (GA) through nasal endotracheal intubation. Pertinent landmarks of the face, such as the corner of the mouth, lower lip, and the entire ear, were left uncovered during the procedure to orient the surgeon to the course of the facial nerve and permit observation of motor function of the lip. The surgical landmarks were marked before the injection of a vasoconstrictor (Figure 2).

**Figure 3:**
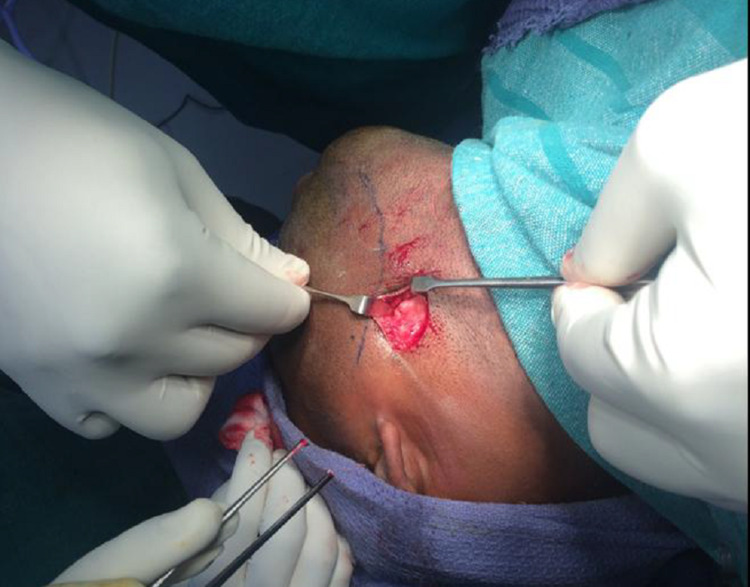
Platysma muscle beneath the subconnective tissue (Patient 1)

The retromandibular transmasseteric approach was used to avoid damage to the facial nerve. The incision was placed 0.5 cm below the earlobe and continued inferiorly for 5 cm. It was placed just behind the posterior border of the mandible and was extended below the level of the mandibular angle, depending on the extent of exposure desired according to the level of the fracture. The initial incision was carried out through the skin and subcutaneous tissues to the level of the platysma (Figure 3) [10].

**Figure 4:**
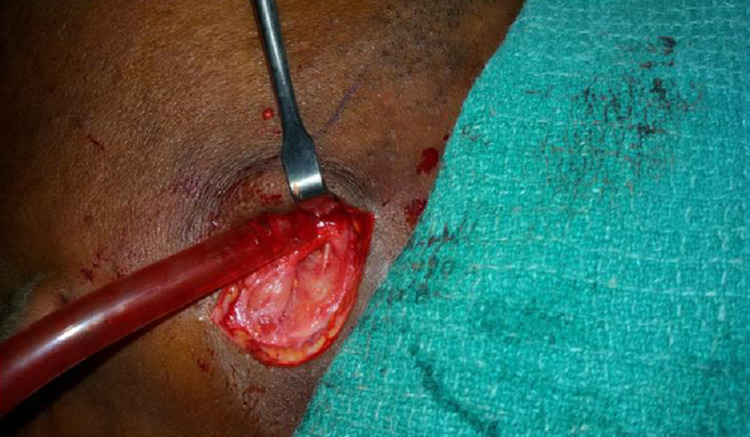
Marginal mandibular branch of the facial nerve (Patient 1)

The platysma muscle and superficial musculoaponeurotic system (SMAS) were incised in the vertical plane. A hemostat was spread open parallel to the anticipated direction of the facial nerve branches. Dissection was carried out and the marginal mandibular nerve was preserved when encountered (Figure 4) [10].

**Figure 5:**
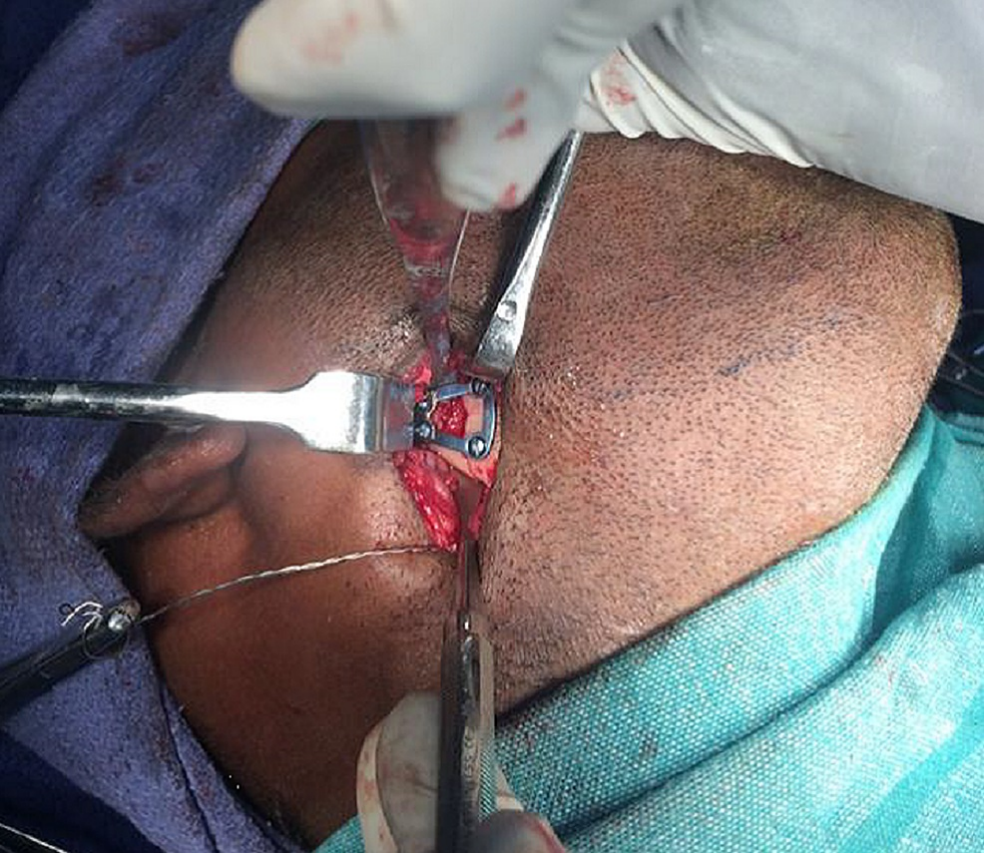
Fixation of three-dimensional (3D) trapezoidal plates to the fractured segments (Patient 1)

**Figure 6:**
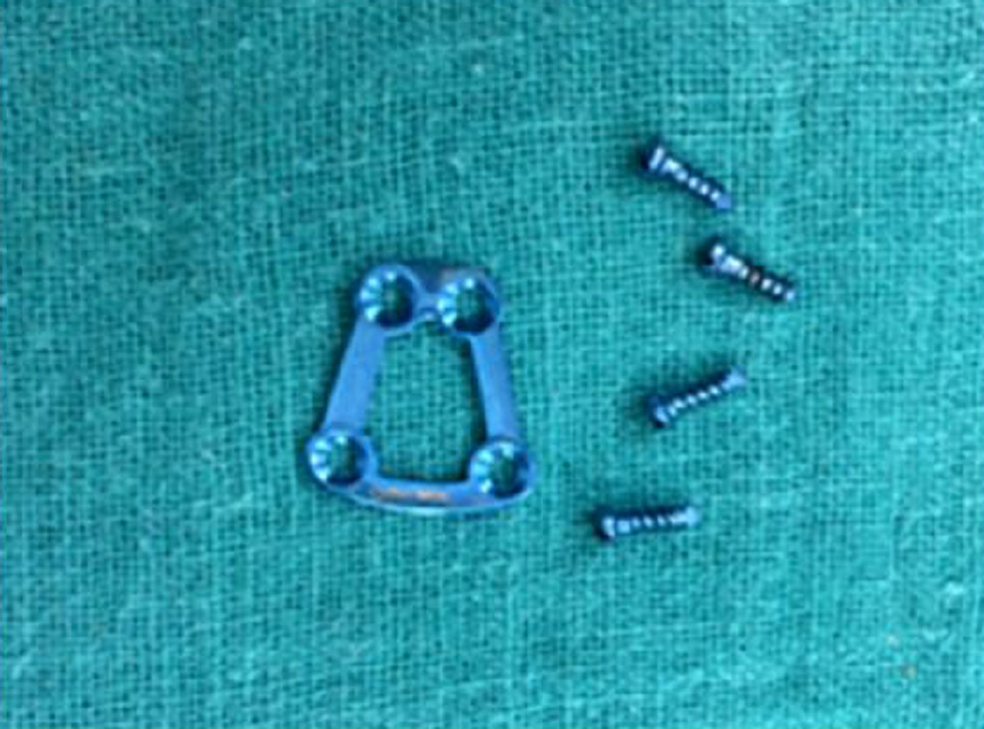
Trapezoidal three-dimensional (3D) plates with screws used in study

A broad retractor was placed behind the posterior border of the mandible to retract the retromandibular tissues medially and the pterygomasseteric sling was incised sharply. After stripping the masseter from the lateral surface of the mandible, the entire lateral surface of the mandibular ramus up to the level of the temporomandibular joint (TMJ) capsule, as well as the coronoid process, were exposed [10]. When using this approach for open treatment of condylar process fractures, it was often necessary to retract the mandibular ramus inferiorly. A simple technique was followed by first applying a bi-cortical bone screw through the gonial angle region (taking care to avoid the inferior alveolar canal) and using a 24-gauge wire to retract the mandible inferiorly, allowing the reduction of fracture fragments. Once the fracture was reduced, the titanium 3D condylar plates (2 mm x 4 holes) were positioned, such that the long arm of the plate was along the posterior border (Figure 5). Titanium screws (2 mm x 6 mm) were used, two on each side of the fractured segment, and the screw over the proximal fragment was placed first (Figure 6).

After checking for occlusion and ensuring the correct position of the condyle in the glenoid fossa, the surgical wound was irrigated with saline and betadine. Any associated mandibular fracture was opened through an intraoral approach and appropriate mini-plates were used for osteosynthesis as per Champy’s principles [6]. Layer-wise closure was carried out with 3-0 vicryl and 4-0 nylon. A pressure dressing was placed.

**Figure 7:**
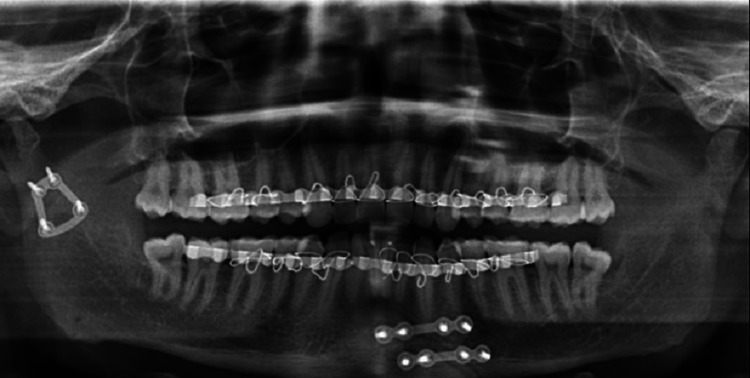
Postoperative OPG showing reduced fractured condylar component fixed with three-dimensional (3D) trapezoidal plate (Patient 1) OPG: orthopantomogram

**Figure 8:**
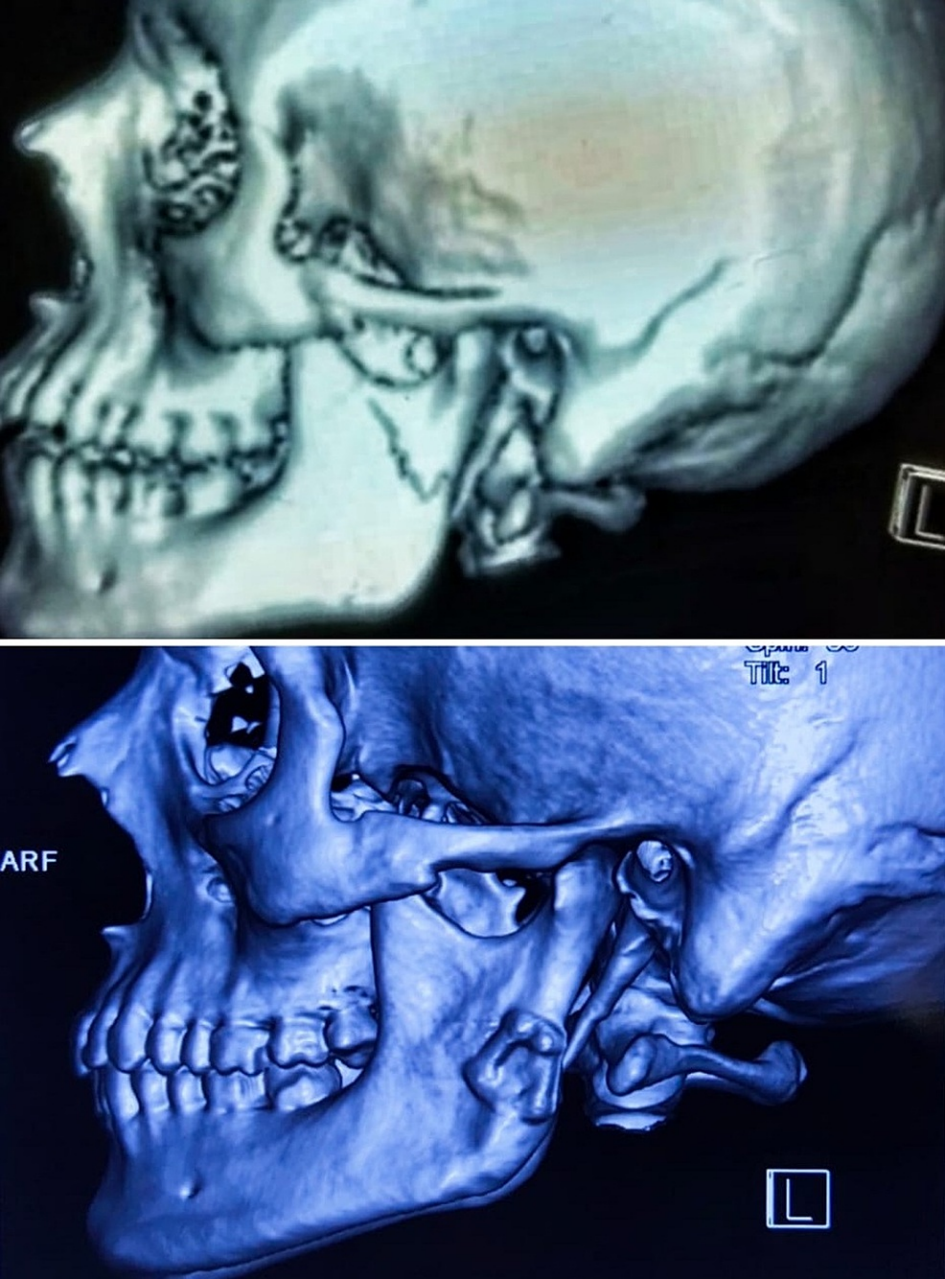
Preoperative and postoperative computed tomography of the facial bones (Patient 2)

The patients were administered postoperative medications which included intravenous antibiotics and anti-inflammatory analgesics for a period of about five days to seven days, as well as maintaining adequate hydration status and nutritional requirements. A soft diet and physiotherapy were started on the first postoperative day. Patients were not kept under postoperative MMF. Sutures were removed between the 7th and 10th postoperative days. All the patients were advised to adhere to a soft diet for about three weeks postoperatively and instructed to maintain good oral hygiene. All patients were followed up postoperatively at one week, one month, three months, and six months. An OPG was taken to assess the osteosynthesis (Figures 7-8).

## Results

Statistical analysis was done using the IBM® Statistical Package for Social Sciences (SPSS) Statistics®, version 21 (IBM® SPSS Statistics® for Windows, Armonk, NY). Chi-square test (Pearson’s), Friedman’s test for repeated measures, and Wilcoxon signed-rank test were done for qualitative data, whereas analysis of variance (ANOVA) was done for quantitative data.

Demographic characteristics

A total of 20 cases with subcondylar fractures of the mandible were operated on. Demographic data revealed that 17 were males and three were females. The mean age of the group was 28 ± 1.22 years. Only five patients had isolated subcondylar fractures. None of the patients treated had bilateral subcondylar fractures as they were excluded from the study. All the cases were followed up regularly up to six months at regular intervals of time and there was no attrition of cases.

Mouth opening

In our study, all patients had a mouth opening of < 30 mm preoperatively, and this increased in all patients to > 40 mm within one month postoperatively (Table 1). The P-value (P < 0.001) was significant.

**Table 1:** Friedman’s Test for Repeated Measures for Mouth Opening Measured Preoperatively and Then Postoperatively at One Week, One Month, Three Months, and Six Months

Variable	Mean Rank	P-value
Mouth Opening Preoperatively	1.20	< 0.001
Mouth Opening Week 1	1.80	
Mouth Opening Month 1	4.00	
Mouth Opening Month 3	4.00	
Mouth Opening Month 6	4.00	

Mandibular deviation

With regard to mandibular deviation, all the patients had a lateral deviation preoperatively (Table 2). At the one-month postoperative follow-up, 16 patients had lateral deviation, which then was reduced to three patients at the end of six months (P-value = 0.000).

**Table 2:** Chi-Square Test for Presence or Absence of Mandibular Deviation in Patients Preoperatively and Postoperatively at One Week, One Month, and Six Months * represents a statistically significant P-value N: number of patients; %: percentage

Chi-Square Test for Presence or Absence of Mandibular Deviation					
Mandibular Deviation	Preoperative		One Week Postoperative		Chi-square P-Value
	N	%	N	%	1.000
Present	20	100	20	100	
Absent	0	0	0	0	
Mandibular Deviation	Preoperative		One Month Postoperative		Chi-Square P-Value
	N	%	N	%	0.106
Present	20	100	16	80	
Absent	0	0	4	20	
Mandibular Deviation	Preoperative		Six Months Postoperative		Chi-Square P-Value
	N	%	N	%	
Present	20	100	3	15	0.000*
Absent	0	0	17	85	

Occlusal derangement

All the patients had occlusal derangement preoperatively. During the first postoperative week, 11 patients had mild occlusal discrepancies. By the end of three months follow-up, only three patients had mild occlusal discrepancies (P-value = 0.000) (Table 3).

**Table 3:** Chi-Square Test for Presence or Absence of Occlusal Deviation in Patients Preoperatively and Postoperatively at One Week, One Month, and Six Months * represents a statistically significant P-value N: number of patients; %: percentage

Chi-Square Test for Presence or Absence of Occlusal Deviation					
Occlusal Deviation	Preoperative		One Week Postoperative		Chi-square P-Value
	N	%	N	%	0.001*
Present	20	100	11	55	
Absent	0	0	9	45	
Occlusal Deviation	Preoperative		One Month Postoperative		Chi-Square P-Value
	N	%	N	%	0.106
Present	20	100	16	80	
Absent	0	0	4	20	
Occlusal Deviation	Preoperative		Six Months Postoperative		Chi-Square P-Value
	N	%	N	%	
Present	20	100	3	15	0.000*
Absent	0	0	17	85	

Surgical access and plate adaptability

The surgical accessibility and plate adaptability were evaluated subjectively by the operating chief surgeon. They were graded as excellent, good, and fair. In 40% of patients, the accessibility was excellent and 40% had good accessibility. The remaining 20% had only fair access due to a more medial displacement of the condylar fragment. In 80% of patients, the adaptability was excellent; in 20%, it was good due to the presence of severely displaced fractures in the subcondylar region. None of the patients had poor adaptability issues.

Reduction of fractured fragments

The displacement of the fractured condyle in the pre-treatment OPG was measured as the angle between a line drawn between the medial and lateral poles of the condyle and a tangent through the lateral surface of the mandibular ramus. The difference between the angle on the non-fractured and the fractured sides was used as a measure of displacement and assess the reduction of fractured fragments [11]. Comparison of pre-treatment angulation with postoperative angulation revealed a P-value < 0.001. The mean angulation of the reduced condyle postoperatively was 0.00500 ± standard deviation (SD) of 0.420) (Table 4).

**Table 4:** Wilcoxon Signed-Rank Test for Assessment of Reduction of Fracture by Measuring Angulation of Fractured Condyle with Ramus Preoperatively and Postoperatively SD: standard deviation

Variable	Mean	SD	Z statistic	P-value
Angulation Preoperatively	21.8650	2.72344	-3.92	< 0.001
Angulation Postoperatively	0.0050	0.42112		

In the OPG, the reduction of the ramus height was measured by the difference in length between the fractured and non-fractured sides. The radiographic magnification scale was set to 100% in all cases, both in preoperative and postoperative OPGs. The ramus height was calculated as a distance on the ramus tangent between the broadest part of the condylar head and the most inferior contact of the angle of the mandible. The average postoperative shortening of the ascending ramus height compared with the non-fractured side was -0.088 mm with a standard deviation of 0.658 (P-value < 0.001) (Table 5).

**Table 5:** Wilcoxon Signed-Rank Test for Assessment of Reduction of Fracture by Measuring Ramus Length and Angle Preoperatively and Postoperatively SD: standard deviation

Difference in ramal height	Mean	SD	Z statistic	P-value
Preoperative	8.430	1.98	-3.92	< 0.001
Postoperative	-0.088	0.658		

Postoperative pain, scars, and complications

The postoperative pain was measured using the Visual Analog Scale (VAS) from 0 to 10. During the first postoperative week, 66.7% of the patients experienced moderate-intensity pain. At the end of six months, 15 patients were completely pain-free on maximum mouth opening, lateral excursion, and protrusive movements. The results were statistically significant with the P-value < 0.001 done by Friedman’s test for repeated measures. Operative time was measured in minutes, starting with the incision to the closure of the wound. The preoperative time varied with the presence of associated fractures of the facial region; hence, it was not a good variable to study. The average preoperative time was 58.6 min with an SD of ± 10.98 min. The scar was conspicuous in all patients during the first postoperative month and became inconspicuous by six months in four patients. None of the patients had a hypertrophic scar. With regard to complications, four patients had a postoperative hematoma which subsided by one week using non-surgical management.

Wound infection, facial nerve weakness, and osteosynthesis device failure

Wound infection was assessed by the presence of discharge/pus from the surgical site. Two patients had a wound infection during the first postoperative week which was managed by appropriate oral antibiotics and subsided within five days. Since it was only a superficial infection, no incision and drainage or revision of surgery were required. In three patients, postoperative weakness of the marginal mandibular branch of the facial nerve was observed during active mouth-puckering. This recovered in two patients by the time of the six-month follow-up. None of the patients presented with osteosynthesis device failure during the six-month postoperative period which was evaluated by the sequential OPG. Osteosynthesis device failure was assessed by plate bending, plate fracture, and screw loosening.

## Discussion

Mandibular fractures comprise around 65% - 70% of all maxillofacial trauma cases, with only the mandible being fractured in 50% of those cases. During the last 25 years, condylar fractures comprise 17.5% - 52% of all mandibular fractures. The mandibular condylar process is the most common fracture of the maxillofacial region [12-13]. Due to its peculiar functional and anatomic features, unlike the other joints, fractures of the condyle require skilled management to reestablish the anatomical and functionality of the joint and the harmony of the associated osseous, dental, and soft tissue structures. Open reduction of condylar fractures is one of the most debated topics among maxillofacial and plastic surgeons worldwide. A condylar fracture with loss of vertical ramus height associated with or without dislocation of the disco-condylar unit is now widely accepted as an indication for ORIF. In 1994, Walker described the goals for the management of condylar fractures [14]. These included the pain-free movement of the mandible, good occlusion, and symmetry of mandibular movements. Besides selecting the most appropriate surgical approach, it becomes equally important to select an appropriate osteosynthesis device that can withstand and transmit the functional load applied to it in an anatomically and biomechanically complex area like the condylar region. From the biomechanical point of view, factors such as a buttressing effect, loss of bone in the fracture gap, or presence of comminuted fractures play a considerable impact on the primary and secondary stability of the osteosynthesis device.

The mandible is shown to exhibit numerous complex combinations of movements and torsion patterns, which must be considered when evaluating the stability of the osteosynthesis device [15]. In 2003, Choi et al. reported superior biomechanical stability of the double mono-cortical miniplate technique when compared to other plating techniques for condylar fractures [16]. The main factor affecting the stability of osteosynthesis of the condylar neck are the incongruent reduction of fracture gaps due to inadequate adaptation of plates. The gaps between the fractured segments after reduction have a considerable effect on the stability of the plate. The double mini plating technique, although the most widely accepted and used for reduction of subcondylar fracture, the surgeon is confronted with the problem of restricted visualization of the condylar neck area, which impedes a correct anatomic reduction and plate adaptation. Many authors have reported a high failure rate associated with double plating technique of up to 35 % including plate fractures [13, 17-18].

In 2006, Meyer et al. demonstrated experimentally that the use of double plating in many instances does not provide sufficient strength to withstand the physiological strain in the subcondylar region during function [18]. Literature comparing stabilization hardware shows that adaptation plates are least favorable and the mini-dynamic compression plates are most favorable and provide more stability [19]. Meyer et al. tested three osteosynthesis devices and found that none of the tested devices fulfilled the principles of functionally stable osteosynthesis resulting in instability or plate fracture [6-7, 20]. This became the starting point that led to the development of trapezoidal condylar plates (TCP) - 3D (Medartis AG, Basel, Switzerland), designed specifically for use in low and high subcondylar fractures. The trapezoidal plates are designed following the findings of an in-vitro analysis of load, strain, and bone deformation at the condyle.

During the functional activity, there are tensile strains at the anterolateral border compressive strain along the posterior-medial border causing lateromedial bending of the condyle [4]. These principles mandate that to provide the best possible bio functionality, the plates must be placed along the ideal lines of osteosynthesis. The plate is in a trapezoidal shape so that the anterior arm of the plate can be superimposed over the tension lines under the sigmoid notch. The 3D plates were developed to offer enhanced stability, less periosteal destruction, osteosynthesis of small fragments, minimum osteosynthesis material, and ease of placement.

Out of 20 patients, 15 were having associated fractures of the mandible. In 2010, Sawazaki et al. reported that subcondylar fractures result mainly due to tensile failure via distributed indirect impact leading to extreme bending of the mandibular neck as one of the weakest points in the mandible [20]. This mechanism explains the correlation between other fractures of the mandible and condylar fractures. The mean amount of maximal active interincisal opening was 41.6 mm (range: 30 to 60, SD ± 2.1 mm). This value was acceptable in comparison to Landes and Sader’s study in 2007 [21]. Measuring lateral movements of the mandible are better indicators than mouth opening in evaluating the functional ability of TMJ because these measurements are ideal for assessing translational motions of the condyle which could be affected to a greater degree through the damage of TMJ caused by fractures [22]. In this study group, the lateral protrusive movements were within normal range without significant difference between the fractured and non-fractured side by the third postoperative month, consistent with the results of another study by Trost et al. in 2009 [23].

The challenge in treating subcondylar fracture is to restore the pre-traumatic occlusal relationship. In the literature, percentages of post-ORIF malocclusion vary widely. This may be attributed to the patient’s previous dental status, presence of an additional fracture in the maxillofacial region, bilateral condylar fractures, or inadequate reduction of the fractured fragments [24]. Occlusal disturbances were noted at six months follow-up in only three out of 20 patients, consistent with the findings of Ellis et al. in 2000 [25]. Accessibility is measured by the ability to reduce the fracture and the ease of placement of plates and screws. A retromandibular transmasseteric approach was used which provided good surgical access to the subcondylar region, consistent with the results of Biglioli et al. in 2009 [26]. All functional parameters show significantly better outcomes in patients with a subcondylar fracture treated with open reduction and internal fixation [27].

In this study, it was found that it is easier to adapt the 3D plates in the subcondylar region along the lines of osteosynthesis which may be attributed to its geometric configuration. Reduction of the fracture after placement of the 3D plates was also excellent to good, which was similar to reports by Meyer et al. [24]. Radiographic evaluation demonstrated good accuracy of anatomic reduction in all cases. Postoperatively, the mean difference in ramus height was - 0.088 mm ± SD 0.658 mm and angulation 0.005 ± SD 0.420. Four patients had unaesthetic scars. In 2000, Ellis et al. reported unaesthetic scars in 7.5% of the cases in their cohort of 93 patients [28]. In 2008, Meyer et al. reported a wound infection rate of 1.45%. In our study, two patients had a superficial wound infection which may be attributed to contamination from the intraoral wound site despite the aseptic precautions taken. One patient had weakness of the marginal mandibular nerve at six months postoperatively. This may be due to a retraction injury during surgery. None of the patients reported clicking or TMJ pain at the end of six months. There was no radiographic evidence of plate bending, screw loosening, or plate fracture during the six-month follow-up. The geometric form of the trapezoidal 3D plates is designed with the idea to resist forces in three dimensions. The quadrangular shape allows for placing one arm parallel to the condylar axis and the other arm under the sigmoid notch along the lines of osteosynthesis in condyle.

The small sample size and limited follow-up could be considered as the limitations of this study.

## Conclusions

Trapezoidal 3D condylar plates have an anterior and a posterior arm that enables them to provide enhanced biomechanical stability against the tension, compression, and torsion forces encountered at the anterolateral and posterolateral borders of the condylar region of the mandible. It is also feasible to place these plates along Champy's lines of osteosynthesis. Further, the requirement of the osteosynthesis device to achieve this is reduced by half in comparison with the gold standard double miniplate technique for subcondylar fractures.
